# Bovine Ephemeral Fever Viruses in Israel 2014–2023: Genetic Characterization of Local and Emerging Strains

**DOI:** 10.3390/pathogens13080636

**Published:** 2024-07-29

**Authors:** Natalia Golender, Bernd Hoffmann, Gabriel Kenigswald, Shani Scheinin, Maor Kedmi, Dan Gleser, Eyal Klement

**Affiliations:** 1 Department of Virology, Kimron Veterinary Institute, Bet Dagan 5025001, Israel; 2 Koret School of Veterinary Medicine, The Robert H. Smith Faculty of Agriculture, Food & Environment, The Hebrew University of Jerusalem, P.O. Box 12, Rehovot 76100, Israel; dgleser@gmail.com (D.G.); eyal.klement@gmail.com (E.K.); 3 Institute of Diagnostic Virology, Friedrich-Loeffler-Institut, Südufer 10, 17493 Greifswald-Insel Riems, Germany; bernd.hoffmann@fli.de; 4 Hachaklait Veterinary Services, Caesarea 3088900, Israel; kenigswald@hak.org.il (G.K.); scheinin@hak.org.il (S.S.); kedmi@hak.org.il (M.K.)

**Keywords:** cattle, epizootic, outbreak, phylogenetic analysis

## Abstract

Bovine ephemeral fever (BEF) is an arthropod-borne viral disease, which frequently causes significant epizootics in susceptible water buffalo and cattle in Africa, Australia, Asia and the Middle East. In the current study, a two-stage protocol for BEFV viral isolation was developed. Data on the clinical signs, geographic distribution and phylogenetic analysis of BEFV strains isolated in Israel in 2015, 2018, 2021 and 2023 were summarized. It was found that during 2015–2021, all BEF outbreaks were caused by local BEFV strains, whereas the epizootic of BEFV in 2023 was caused by a new “Mayotte-like” BEFV strain. A comparison of bluetongue (BT) and BEF outbreaks during 2023 in Israel demonstrated that the incidence of BEFV was 2.21 times higher and its pathogenicity was more serious for the cattle population compared to that caused by BTVs. A phylogenetic analysis of Israeli and global BEFV revealed the emergence of non-local strains in new areas. This finding suggests that BEFV can no longer be classified based only upon geographic distribution. Considering a phylogenetic, genetic and proteomic analysis of all available BEFV strains, we suggest classifying them as a single serotype, which includes four lineages.

## 1. Introduction

Bovine ephemeral fever (BEF) is an arthropod-borne viral disease that is suspected to be transmitted by mosquitoes and *Culicoides* biting midges [1]. According to serological studies, BEF viruses infect a large range of domestic and wild ungulates, while cattle and water buffalo are considered the most clinically susceptible animals. Clinical disease was also observed in yak in India and China [2,3,4,5]. BEFV was successfully isolated from insects that were not recently blood-engorged. These insects included both midges and mosquitos consisting of several different *Culicoides* species (Diptera: Ceratopogonidae)*,* some species of mosquitos (Diptera: Culicidae), such as *Anopheles bancroftii,* and a mixed pool of mosquitoes that included species from the genera *Culex*, *Uranotaenia* and *Aedes* [2].

BEFV (species *Ephemerovirus febris*) belongs to the *Rhabdoviridae* family and *Ephemerovirus* genus. The virion contains a ~14.9 kb long, non-segmented, negative-sense single-stranded RNA genome that encodes five structural proteins—nucleoprotein (N), phosphoprotein (P), matrix protein (M), glycoprotein (G) and RNA-dependent RNA polymerase (L)—and five non-structural proteins (NSs) (Gns, α-1, α-2(3), β, γ) [6,7]. Among the structural proteins, G plays a crucial role in the attachment, entry and release of viruses. It contains highly conserved immunodominant epitopes, stimulates the production of host-neutralizing antibodies and is regarded as one of the major antigenic determinants [8,9].

The morbidity rate in susceptible cattle populations is usually high (might reach 80–100%), whilst the mortality rate, in most cases, remains low (1–2%) [10]. The duration of clinical signs is usually short, characterized by bi-phasic acute hyperthermia, lameness and ocular and nasal discharge before recovery [2]. In Mayotte, BEF in cattle (locally named “cattle flu”) has been associated with anorexia, nasal discharge, hyperthermia and lameness [11]. In more seriously affected cattle, recumbency, synovitis, lethargy, muscle stiffness, lameness, reluctance to move, inappetence, muscle stiffness, anorexia, ataxia, paralysis and death may be reported. Moreover, neurotropism was confirmed by a histopathological examination of the brain, spinal cord and peripheral nerves and may causally contribute to paresis or paralysis caused by BEF [12,13].

However, during the last two decades, there have been reports indicating the increasing frequency of reappearing and severity of the disease with alarmingly high case fatality rates, sometimes exceeding 20% in China and Turkey [14,15,16]. In Israel, according to previous publications, case fatality rates were higher in milking cows as compared to heifers. Thus, the highest case fatality rate was registered in 1999, ranging from 8.8% in heifers aged from 1 year to calving and up to 11.5% in cows [17]. Regarding the last described outbreak, dated 2021, the culling range in clinically diagnosed cows in different farms was between 0 and 15.9%, with an average fatality rate of 4.83% [18]. Notably, experimental infection also causes clinical illness, characterized by hyperthermia, nasal mucus discharge and lacrimation, and some “serious” clinical signs of BEF [19].

BEF has a major economic impact, especially on dairy herds. Economical losses are linked with the reduced milk production, death and culling of seriously affected animals in the dairy industry, while it is linked to a loss of production in the feedlot cattle industry, to temporary infertility in males and to the temporary disablement of draught animals [1,18].

There are documented cases of BEF dated 1906 in Zimbabwe, when an epizootic of the disease was first observed. Since then, the disease has been commonly reported in many other sub-Saharan African countries, including South Africa, Sudan, Kenya, Uganda and Tanzania [1,20]. However, historical reports suggest that the regional distribution of BEF in Africa is probably larger, including Madagascar, Mayotte and the Indian Ocean region [11]. Outside of Africa, the disease occurs over a vast global area; the Middle East, South and South East Asia throughout most of China, extending into Taiwan, the Korean Peninsula and southern Japan, into northern and eastern Australia, and becoming endemic in these regions [1,11,21].

In the Mediterranean region, BEF was first described in Egypt in 1924 [22,23] and in the Jordan Valley of Palestine in 1931 [24]. The disease has also been reported in Jordan, Syria, Iraq, Iran and Saudi Arabia [25,26]. In recent years, BEFV was registered in Turkey (2008, 2012, 2020), [16,27,28], Iran (2013, 2018, 2020) [29,30] and Egypt in 1991, 2000, 2001, 2004 [31] and 2017 (source—GenBank, accession number (acc. no) MH939257).

In Israel, the disease was first reported in 1951, reappearing irregularly with long intervals (1990, 1999, 2004) [17]. Serological testing of subclinical calf (sentinel) sera collected during the inter-epizootic period 2006–2007 revealed no positive serum samples, which suggests no exposure to the virus after 2004 and until its probable re-invasion from Turkey in 2008 [32]. Following this, several outbreaks emerged and were registered in 2010–2011, 2014–2015, 2017–2018 and 2021 [18]. From June until December 2023, an epizootic of BEF occurred again, affecting most geographical areas in Israel. This epizootic was associated with the Mayotte-like BEFV strains, which had never been detected in Israel previously.

Bluetongue (BT), similar to BEF, is an arthropod-borne viral disease of domestic and wild ruminants with a world-wide distribution. The transmission of most BTV serotypes between mammalian hosts relies on competent blood-feeding midges of the *Culicoides* species [33]. In contrast to BEF, BT is a disease reportable to the WOAH [34]. The long-term infection of cattle was thought of as subclinical, with the exception of BTV-8 infection. Cattle are particularly significant in the epidemiology of the disease due to the prolonged viremia that occurs following infection. When apparent, the clinical signs of BT are mainly attributable to an increase in vascular permeability and include fever, hyperemia and congestion, facial edema and hemorrhages, erosion of the mucous membranes, coronitis, laminitis and pleural and pericardial hemorrhages [33]. The impact of the current outbreaks in Europe caused by serotype 3 in 2024 in cattle is currently being investigated by GEZONDHEIDSDIENST VOOR DIEREN BV(GD), Netherlands [35]. For several years, the influence of different BTV serotypes on the health of cattle in Israel has been investigated. It was demonstrated that BTV-1, -3, -5, -6, -8, -9, -12, -15 and -24 caused clinical manifestations of BT in Israeli cattle. For example, BTV-9-infected milking cows manifested hypersalivation, fever, dyspnea, recumbency, milk reduction and diarrhea [36,37].

BTV belongs to the genus *Orbivirus* within the family *Sedoreoviridae*. Unlike the linear BEFV genome with a single serotype, the genome of BTV is composed of 10 linear double-stranded segments (Seg-1 to Seg-10) encoding seven structural (VP1 to VP7) and five nonstructural (NS1 to NS5) proteins [38]. Based on Seg-2 gene sequences and virus neutralization tests, 36 distinct BTV serotypes have currently been officially recognized [39,40].

In this article, we describe the clinical manifestation, spread dynamics and duration of the BEFV 2023 outbreak in specific farms and across the entire Israeli territory. We analyzed the data collected from affected cattle and tested in parallel for BTV and BEFV, which enabled us to compare and evaluate both of the pathogens’ veterinary importance in context of the 2023 outbreaks. In addition, we molecularly characterize the Israeli BEFV strains collected during the outbreaks in 2015, 2018, 2021 and 2023 and show that this outbreak was caused by viruses which are similar to strains isolated previously in Mayotte. Our findings suggest the emergence of a virulent BEFV strain in the Middle East.

## 2. Materials and Methods

### 2.1. Field Samples

A total of 1176 samples from 1174 cattle were collected and submitted to the Kimron Veterinary Institute’s Department of Virology, Israel, between May and December 2023 for routine examination of arboviral infection in Israel. Clinical specimens comprised whole blood from clinical animals, and brain, lung or/and spleen from dead or severely ill slaughtered cattle. The field samples which were tested in parallel for BEFV, BTV and epizootic hemorrhagic disease virus (EHDV) from cattle are shown in Table 1. Since EHDV did not cause an outbreak in 2023 and was previously published, this information was not considered in this study [41].

### 2.2. Viral Isolation (VI)

Most BTVs were isolated in embryonated chicken embryos (ECEs) according to Komarov et al. [42] followed by adaptation on cell cultures (Table 2). For VI, red blood cells were washed three times with PBS and disrupted with sterile double-distilled water in a proportion of 1:10, and the supernatant from homogenized spleen filtered with a 0.22 µm filter was used for BTV VI. The information on VI from field cattle is presented in Table 1. Additionally, samples from sheep, six whole blood samples and one spleen sample were used for BTV VI (data are not presented in Table 1).

**Figure 1:**
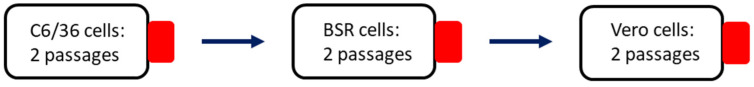
Scheme of the final protocol of the developed method for the isolation of BEFV.

Attempts to isolate BEFV that were performed in ECEs and directly in Vero (origin: green monkey kidney) and BHK-21 (origin: hamster kidney) cell cultures failed. The following protocol was developed for the isolation of BEFV: (i) primary infection of the 24 h monolayer of C6/36 cells (origin: *Aedes albopictus*) with washed buffy coats for 2 passages with confirmation of VI by RT-PCR [43,44]; (ii) infection of the 24 h monolayer of the BSR cells (BHK-21 clone BSR) with BEFV-positive supernatant from C6/36 cells with continuous re-passages; and (iii) infection/adaptation of the 24 h monolayer of Vero cells with the RT-PCR BEFV-positive BSR cell supernatant with a prominent cytopathic effect. The final schematic protocol for BEFV isolation is shown in Figure 1.

Washed buffy coats or buffy coats together with red blood cells (“infection substance”) were used for VI. Thus, the infection substance collected in 2018 was stored at −70 °C, whereas the freshly prepared infection substance collected during the 2021 and 2023 arboviral period (June–December) was stored +4 °C. C6/36 culture cells were incubated at 30 °C for two hours with the infection substance. Thereafter, the infection substance was collected and the cell culture was carefully washed twice with PBS. Infected C6/36 cells were maintained in media containing Leibovitz’s L-15 tissue culture medium, containing 2% of fetal calf serum (FBS), 1% L-glutamine and 1% penicillin–streptomycin (10,000 U/mL). The infected C6/36 cells were incubated for four to six days depending on the cell culture condition. Between every passage, the infected cells were stored at −70 °C. After two passages in C6/36 cells, BSR cells were exposed to the BEFV-positive supernatant from C6/36-infected cells for two hours in the cell incubator at 37 °C. Infected BSR cells were incubated in the cell incubator at 37 °C for between four and six days depending on the cell culture condition. For adapting local BEFV strains to the BSR cells, demonstrating a prominent cytopathic effect (CPE), usually two passages were needed. However, in the case of the “Mayotte-like” BEFV strains, in most cases only a single passage was needed, as well as for the adaptation from BSR to Vero culture cells. The infected Vero culture cells were incubated in a cell incubator at 37 °C for between five and seven days. Infected BSR and Vero cells were maintained in media containing Dulbecco’s Modified Eagle’s Medium (DMEM) and supplemented with 2% FBS, 1% tryptose phosphate broth and 1% penicillin–streptomycin (10,000 U/mL). Table 2 shows a schematic description of the different procedures used for BEFV and BTV isolation.

Because the infectious material was collected and the infected cells being washed, almost no infectious material remaining on the surface of the cells and in the supernatant/tissue culture medium, making subsequent sequencing unnecessary. In the majority of cases, a positive result from successful VI was strongly positive in RT-PCR, which confirmed VI. In cases of infected C6/36, where a prominent CPE was rarely seen, PCR was performed for every infected case after two blind passages. Only RT-PCR-positive C6/36 supernatants were used for the infection of the BSR cells.

### 2.3. Nucleic Acid Extraction and Polymerase Chain Reaction (RT-PCR)

Ribonucleic acid (RNA) from the cell culture supernatant and field samples (whole blood, lung, brain, spleen) were extracted with the MagMAX™ CORE Nucleic Acid Purification Kit (Thermo Fisher Scientific, Austin, TX, USA) or IndiMag Pathogen Kit (Indical Bioscience, Leipzig, Germany), according to the recommendations of the manufacturers. During the arboviral period (June–November 2023), all samples of cattle origin were tested for BTV and EHDV according to Wernike et al. [45]. Brain samples from cattle which manifested neural signs before death or euthanasia were also routinely tested for simbuviruses [46]. The laboratory diagnosis of BEFV during the outbreak of 2023 was performed based on pan-ephemero conventional RT-PCR [44], followed by the Sanger sequencing of selected samples.

### 2.4. Sequencing and Phylogenetic Analyses

BEFV-positive field samples (plasma or serum) collected in 2015 were partially sequenced by the Sanger method using overlapping genome fragments (the primer sequences are presented in Table S1). The cDNA fragments were purified with the MEGAquick-spin Total Fragment DNA Purification Kit (iNtRON Biotechnology, Gyeonggi-do, Republic of Korea) and subsequently sequenced at Hylabs, Rehovot, Israel, by standard Sanger methods in both directions using an ABI 3730xl DNA Analyzer (Thermo Fisher Scientific).

The whole-genome coding regions of BEFV genomes were sequenced as previously described [44]. For the sequencing of the strains collected in 2018 and 2021, the buffy coats of cattle from field samples were used, while for the strains from 2023, the sequencing was performed for viral strains isolated in BSR cells. The extracted RNA was submitted to the Technion Genomic Center (Technion Israeli Institute of Technology, Haifa, Israel) and was sequenced using an Illumina NextSeq2000 together with a P1 flow cell run in 2 × 150 bp mode (Illumina, San Diego, CA, USA). The sequences were mapped to the most closely related strains (local Israeli strain, acc. no MN078236, and a strain from 2023 from Mayotte, acc. no MN148803). The resulting nt sequences were assembled, aligned and pairwise compared using Geneious version 9.0.5 (Biomatters, Auckland, New Zealand) and/or BioEdit programs (https://bioedit.software.informer.com/7.2/, accessed on 10 July 2024). Phylogenetic trees were constructed using the Mega X software [47]. For all phylogenetic trees, the maximum likelihood method (ML) and the Tamura–Nei models were applied.

### 2.5. Analysis of BEFV Proteins and Noncoding Regions of the Viral Genome

We analyzed all viral proteins and intergenic noncoding regions (ncr) except for 3′ UTR and 5′ UTR, because some publicly available BEFV genomes and BEFV strains sequenced during this study are incomplete at the genomes’ ends. In this analysis, we included all completely sequenced Israeli strains (coding regions; isolates from 2018, 2021 and 2023), two partially sequenced strains from the field samples collected in 2015 and selected publicly available genome sequences of BEFV strains representing every lineage.

## 3. Results

### 3.1. Clinical Disease Manifestations of Affected Animals and Geographic Distribution of BEFV during 2023 Outbreak

In June 2023, a disease clinically resembling BEFV emerged in about twenty milking farms situated in the southern areas of the country (the Arava and Negev desert areas). In most cases, milk reduction for a couple of days preceded the appearance of other clinical manifestations. Thereafter, affected cattle manifested signs which included fever, lameness or stiff gait, recumbency, weakness, hypersalivation and mucosal hyperemia, inappetence, dyspnea or tachypnea. In rare cases, weight reduction, constipation or diarrhea were also seen. According to the reports of clinicians, the case mortality rate was lower than in cases of BEFV-infected cows from previous outbreaks, when the BEF was caused by local BEFV strains. After recovering from BEF, an unusually high proportion of dairy cows were unable to produce milk at the same level as before the disease. Notably, a massive spread of the BEFV to the central and northern areas of the country was mostly seen from August to October 2023. Another exceptional characteristic of the 2023 BEFV epizootic was the recurrent waves of infection or prolonged infections, which were seen in previously affected farms, probably in other previously non-infected animals. Thus, in several farms/geographic settlements, laboratory-confirmed new cases were registered during several months or at long intervals (Table 3).

In November–December, the number of BEFV cases constantly decreased. Considering the whole BEF outbreak in 2023, disease was observed across almost all of Israel from the Arava Desert to the Golan Heights and lasted from June to November/December 2023. Eventually, BEFV infection was laboratory-confirmed in 151 geographic settlements.

### 3.2. Comparison of Collected Data about BTV and BEFV Infection in Cattle in 2023 Arbovirus Season

We compared the number of registered BEFV and BTV cases in Israeli cattle in 2023 (the registered BTV serotypes during the 2023 arboviral season were BTV-3, -4, -5, -6 and 8). Thus, 221 BTV-positive field samples were detected, versus 489 BEF from 1109 tested samples of cattle origin, which were tested for both pathogens (an additional 17 BEFV-positive samples were found among 67 serum samples submitted for BEFV laboratory diagnosis) (Table 1). Considering only the samples which were tested in parallel for BTV and BEFV, the number of BEFV detections during 2023 was 2.21-fold higher than the number of BTV detections.

### 3.3. Viral Isolation

Data regarding BTV VI from cattle origin only are presented in Table 1. In general (both from cattle and sheep field samples), sixteen BTVs were isolated: three strains of BTV-3, four strains of BTV-4, five strains of BTV-5, three strains of BTV-6 and one strain of BTV-8. Thirteen BTVs were isolated in ECEs. Two BTV-3 strains were isolated in cell cultures from whole blood samples from sheep: one in C6/36 cells, and one in BSR. One BTV-4 from cattle whole blood sample was isolated directly in cell cultures.

Data on successful VI for BEFV are also presented in Table 1. Notably, VI of the BEFV from selected samples collected in November and December 2023 was successful from the earliest collected whole blood sample (mid-November 2023), which could point to the last “active” cases dating to mid-November. From the same BEFV/BTV-positive whole blood sample, a Mayotte-like BEFV was isolated directly in C6/36 and BSR cell cultures, while BTV-4 was isolated only in ECE. This result shows the different susceptibility of different VI systems to BTV and BEFV and that a simultaneous infection of a sick female calf with BTV/BEFV could be confirmed. Information on BTV VI from sheep samples is not provided in Table 1.

Table 2 shows that attempts to isolate BEFV directly in ECE, BHK-21 and Vero cells have failed, in contrast to successful direct isolation/adaptation in C6/36 and/or BSR. The proportion of successful VI of the BTV in ECEs was significantly higher than in the cell cultures used for viral isolation in the current work.

### 3.4. Sequencing and Phylogenetic Analysis

**Figure 2:**
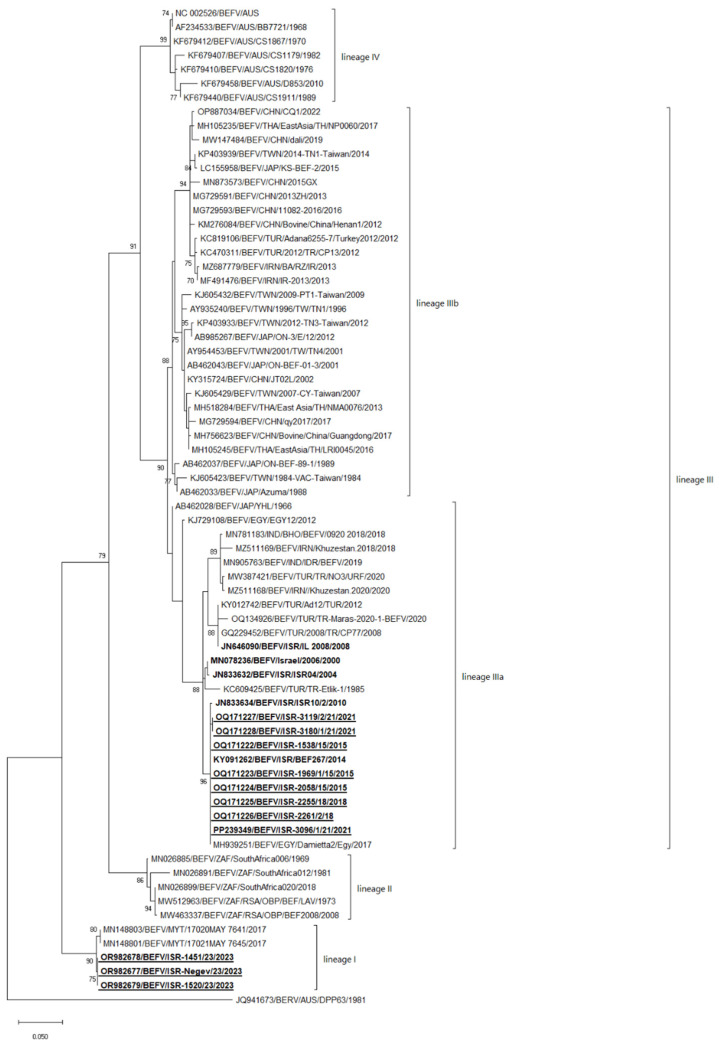
Phylogenetic tree of Israeli and global bovine ephemeral fever viruses (BEFVs) analyzed by glycoprotein-coding nucleotide sequences. The Berimah virus was used as an outgroup. All Israeli BEFV strains are shown in bold, while the strains sequenced during the current study are shown in bold and underlined. The phylogeny was inferred using the maximum likelihood method and the Tamura–Nei model method. The percentages of replicate trees in which the associated taxa clustered together in the bootstrap test (1000 replicates) are shown next to the branches. Viruses were identified by accession number/virus species/location/isolate/year.

The coding regions of the two BEFV strains from 2018 (ISR-2055/18 acc. no OQ171225, ISR-2261/1/18 ac. no OQ171226), three strains from 2021 (strains ISR-2096/1/21, ISR-3119/2/21, ISR-3180/1/21; acc. no PP239349, OQ171227, OQ171228, respectively) and three strains from 2023 (ISR-1451/23, ISR-Negev/23, ISR-1520/23, acc. no OR982678, OR982677, OR982679, respectively) were sequenced. Since the number of completely sequenced global BEFVs is scarce and did not allow us to evaluate epidemiological aspects based on these data, we used the G protein-coding region for phylogenetic analysis (Figure 1). In addition, we provide a phylogenetic analysis based on the publicly available data of the full genome in Figure S1. Before the detection of BEFV strains in Mayotte in 2017 (date of publication 2019), BEFVs were categorized into three or four clusters according to their geographical distribution [2,14,30,48]. In addition, non-local newly introduced BEFV strains are frequently identified in BEFV endemic areas, and so a geography-based classification is already less relevant. For this reason, we divided BEFVs into four lineages (I-IV) based on the phylogenetic relationship between strains, the structure of the proteins and the intergenic regions (Figure 2).

Since no recombination was observed among the BEFV strains and the phylogenetic comparison of the G-protein gene sequences with the complete BEFV genomes demonstrated the same results (Figure 2 and Figure S1), we used the sequences of the G-protein-coding regions for further analyses, as their number was significantly higher than that of the complete genome sequences. In general, according to this classification, lineage I consisted of the Mayotte and Mayotte-like Israeli BEFVs from 2023. Lineage II consisted of South African strains only. Lineage IIIa consisted of local Israeli BEFV strains from 2000 to 2021 together with other Middle Eastern strains, including the Turkish and Iranian Khuzestan strains identified in 2018 and 2020, the Indian IDR strain isolated in 2019, and the Japanese YHL strain isolated in 1966. Lineage IIIb includes the Far-East Asian strains (originating in China, Japan, Taiwan and Thailand) and the Iranian and Turkish strains from the 2012–2013 year of isolation, while lineage IV includes the Australian strains only. It can be seen that in the African, Middle Eastern, Indian and Indian Ocean regions, strains from one geographical area are often introduced into another. For example, in 2012–2013, in the Middle East region where the local strains belong to lineage IIIa, strains of the Far Eastern lineage IIIb were introduced (Turkish strains Adana 6255-7/Turkey2012, CP13 and Iranian BA/AZ/IR and IR-2013, Figure 1).

The percentage of the nt identity between strains which belong to the same lineage/sublineage, and between strains which belong to different lineages, calculated by the pairwise analysis is presented in Table 4. Two BEFV strains were not used for pairwise analysis due to the boundary results: the Japanese strain YHL isolated in Japan in 1966, acc. no AB462028, and the Egyptian strain EGY12 isolated in 2012 with the acc. no KJ729108, which contradicted the results in the pairwise and phylogenetic analyses and were classified as strains belonging to linage III (Figure 1). In general, the identity between lineages I and II, I and III, I and IV, II and III, and II and IV is between 86.34 and 88.01%. The identity between lineages III and IV is slightly higher at 89.30–91.89%. Inside the same lineage (including sublineage IIIa), the identity between strains is higher than 96%, except for sublineage IIIb, wherein the lowest identity is 94.93%. The identity between strains which belong to the entirety of lineage III ranges between 91.13 and 99.73%.

According to phylogenetic analysis, non-local strains have been detected twice in the territory of Israel since the 2000s: the Turkish strain in 2008 (lineage IIIa) and the Mayotte strain in 2023 (lineage I) (Figure 2).

### 3.5. Analysis of the BEFV Proteins and Noncoding Regions of the Viral Genome

All viral proteins from all lineages have the same length, except for the length of some β-proteins. Thus, the length of most BEFV β-proteins is 147 amino acids (aa). In contrast, the length of the Chinese Henan1 strain isolated in 2012 and the Australian BB7721 strain isolated in 1968 is 107 aa, and the South African RSA/OBP strain is 148 aa long. Interestingly, the length of the intergenic noncoding regions is variable between different lineages, as well as inside the same viral lineage and differences in their length are presented in almost every intergenic region (Table 5). Moreover, the length of all of the noncoding intergenic regions of BEFV strains belonging to lineages III and IV is the same, except for the region between the P and M proteins, where the lengths are 57 and 56 nt, respectively. Lineages I and II differ in the length of noncoding intergenic regions and differ from lineages III and IV, illustrating the diversity of strains of “African” origin.

**Figure 3:**
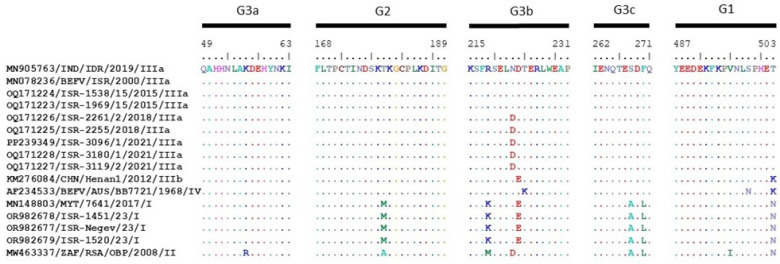
Alignment of the amino acid sequences corresponding to the antigenic sites G1, G2 and G3 of the BEFV G protein of the Israeli and representative BEFV strains. The substitution residues differing from the top sequence are denoted. The BEFV strains used for the analysis are denoted by accession number/country/strain/year of isolation/lineage.

We considered the aa alignment of the antigenic sites of the G protein presented by representative BEFV strains from every lineage and compared them with the sequenced Israeli strains. Thus, all local Israeli strains and the representative Indian strain of the lineage IIIa from 2019 have identical G1, G2 and G3a antigenic sites, while the last Israeli local strains detected in 2018 and 2021 had a single substitution in the G3b site position N222D. The representative Chinese strain Henan1 from 2012, which belongs to the sublineage IIIb, has two substitutions at positions D223E and T503K. The representative Australian strain from lineage IV has two different substitutions from lineage IIIa at positions T224K and S499N, and an additional substitution, N222D, with the local Israeli BEFV strains from 2018 to 2021. Regarding the differences between the sublineage IIIb and the lineage IV, there are three substitutions between the strains: D223E, T224K and S498N. Notably, strains belonging to the sublineage IIIb and the lineage IV have the same aa substitution at the position 503 (K—Lysine), separating them from all other strains belonging to linages I and II (N- Asparagine) and IIIa (T—Threonine). Considering all BEFV strains which belong to lineage III, they are most closely related to the strains belonging to lineage IV (the difference is two to three substitutions). The BEFV strains from Mayotte and Israeli “Mayotte-like” strains (lineage I) have the same antigenic sites, which differ in six positions from the antigenic sites of the BEFV strains from lineage III. The African representative strain (lineage II) differs from the BEFV strains which belong to lineage III in seven/eight positions, while the difference between strains belonging to lineages I and II is five aa substitutions (Figure 3).

## 4. Discussion

Many linear viruses which possess an RNA non-segmented genome, such as, for example, the Newcastle virus, West Nile virus, Rabies virus and Peste der Pestis ruminants, are classified as a single serotype with several lineages/genotypes/clades or classes [49,50,51,52]. Based on genetic, phylogenetic and proteomic analyses, which showed consistent results, we suggest applying a similar nomenclature to BEFV strains. This means classifying all BEFV strains as belonging to a single serotype, which includes lineages I-IV.

A proteomic analysis of the antigenic sites of the G proteins of the Israeli and global strains illustrates that all BEFV strains belonging to sublineage IIIa have almost the same aa sequences. In general, this analysis showed the high similarity of BEFV strains belonging to lineage III (Asian strains) and lineage IV (Australian strains), demonstrating two or three aa substitutions in the antigenic sites of G proteins only. Both phylogenetic and pairwise analyses of the coding regions of G proteins showed similar results illustrating the closest identity between strains belonging to lineages III and IV and far lower identity with strains from lineages I and II (Figure 2 and Figure 3, Table 4). However, multiple substitutions were found in BEFV strains of African and Mayotte origin, which could point to the existence of non-identified additional strains/lineages/sublineages on the African continent and probably to the common ancestor of all BEFVs originating from the African continent.

The introduction of non-local BEFV strains was observed in many geographical areas, except Australia, probably due to the geographic isolation of the continent from other parts of the world. Interestingly, the closest identity of the Australian strains with the Far Eastern strains (sublineage IIIb) points to a probable common BEFV ancestor, while no BEFV exchanges between these regions have been observed for a long period. In contrast, the introduction of new strains to Israel was observed twice during the last 15-year period: in 2008 and recently in 2023. Until 2023, all BEFV outbreaks in Israel were caused by local or regional BEFV strains, which belonged to lineage IIIa, while in 2023, BEFV strains originated from Mayotte, and belonged to lineage I. This fact allows us to presume that the emergence of non-local “Mayotte-like” BEFV strains in Israel can be linked to the uncontrolled movement of infected animals or climate changes, which have influenced wind patterns (affecting the transmission of the viral vector) or facilitated the dissemination of vectors to new habitat areas. Consequently, BEF outbreaks can occur in previously BEFV-free regions.

BEF is a non-reportable disease to the World Organization for Animal Health (WOAH). Nevertheless, the disease’s impact on cattle health and economic losses from the disease can be more serious than those caused by the reportable BT outbreaks. Thus, the reappearance of the BT strains/serotypes, which are responsible for heavy outbreaks among susceptible ruminants, usually happens after relatively long intervals and is probably linked with the long-lived immune status of convalescent or vaccinated animals [33,36,53]. On the other hand, epizootic local BTV strains, which are found annually, are probably less pathogenic and do not cause massive illness, and consequently lead to smaller economic losses to the livestock industry. In contrast, observing the cyclicality of the BEFV outbreaks in Israel, caused by closely related, probable offspring strains of the local BEFV, showed that large outbreaks of BEFV in Israeli as well as in Turkish cattle populations happened in short intervals of 2–4 years (the last registered outbreaks: 2014–2015, 2018 and 2021) [28].

BEFV has been a well-known pathogen in Israel since at least the 1990s. Nevertheless, since the 2000s, when a successful single viral isolation in 2000s was performed in Israel, all efforts to achieve BEFV viral isolation failed. During the current study, an effective two-stage protocol was developed, allowing the isolation of BEFV from field samples in 2018, 2021 and 2023. Effective methods for BEFV isolation can significantly contribute to the production of autogenic live or inactivated vaccines. Autogenic vaccines against homological field strains could have better effectiveness compared with non-homological strains, potentially minimizing the substantial economic impact on the dairy and meat industries.

It was long believed that natural BEF infection typically results in long-lasting immunity [54]. Since the BEF vaccine in Israel is voluntary, and during the past decade several outbreaks have been seen, the dairy herd industry has become increasingly interested in the vaccine. This interest has led to studies evaluating both the short-term and long-term effectiveness of the widely used vaccine based on the 919 strain (Australian strain). Recent studies have demonstrated that the vaccine provides moderate protection, with relatively low neutralizing antibody titers 10–12 months post-vaccination [55,56]. Due to partial vaccination coverage of the susceptible bovine population and the moderate long-term effectiveness of the vaccine, herd immunity in Israel has remained low. This situation is exacerbated by the geographic and climatic conditions favorable to the spread of this arbovirus, leading to frequent BEF outbreaks. Additionally, the annual high replacement rate in local dairy herds, about 25–30%, significantly increases the proportion of susceptible individuals within the herd. This difference between the emerging Mayotte-like BEFV strains and the vaccine strains can probably affect the efficacy of the vaccines used in Israel (the origin of the vaccine strains is lineages IIIb and IV). Short immune protection against re-infection with BEFV can also be linked with a sharp decrease in the antibody level, which dropped more than ten times during only four months from vaccination [57].

Comparative analyses of affected cattle during the 2023 arboviral season showed that in spite of the recognition of multiple BTV serotypes in cattle population, the number of BEFV-affected cattle in 2023 was more than two times higher compared to the BTV-affected cattle despite the fact that the period of RNA detection in the blood of infected animals after BEFV infection is shorter than for BTV infection (the duration of BEFV viremia/RNAemia is probably slightly longer than a week compared to several months of RNAemia for BTV infection) [58]. Consequently, the likelihood of an incidental finding (exposure to the virus without clinical manifestations indicative of disease) is higher in BTV. Therefore, the actual incidence of BEF could be even higher compared to BT, as was determined in the current study. These results illustrate that BEFV was a leading pathogen and the more probable cause of illness in Israeli cattle during the arboviral season in 2023. Unfortunately, it is impossible to evaluate the proportion of BTV- and BEFV-affected cattle from the previous outbreaks because the majority of samples were tested for one pathogen only. Additionally, one of the most important factors driving the epidemiology of BEFV and BTV is a difference in arthropod vectors. According to previous studies, BEFV is transmitted both by midges and mosquitoes [2], while BTV is transmitted by various species of *Culicoides* only [33]. However, vector surveillance in Australia revealed that different species of mosquitos are probably responsible for the most BEFV transmissions [59]. Screening and surveillance programs for studying the abundance of each of these vectors can be extremely important in determining the epidemiology of the two diseases.

In the current study, we demonstrated the significance of BEF outbreaks in Israel and showed that in some years, their economic losses far exceeded those caused by BTV infection in the dairy and beef industries. However, the severity of BT in cattle probably depends on the specific serotype or even strain. Forecasting the next Israeli BEF outbreak is challenging, since BEFV outbreaks in Israel can be caused not only by the local strains, but also by strains which could be introduced into Israel from other regions due to the unique geographic position or replacement of the local strains by the newly emerging “Mayotte-like” strains.

To evaluate cross-virus neutralization data, further serological studies are needed. The absence of this information has complicated the selection of strains for vaccine production and prophylactic strategy, necessitating the close monitoring of vaccine effectiveness and assessing vaccine matches to new strains. The danger of the emergence of BEF in BEF-free areas demands the development of quick and effective diagnostic and preventive tools and an investigation of the pathogen.

## 5. Conclusions

BEFV in 2023 was a major arboviral pathogen in Israeli cattle in spite of the existence of multiple BTV serotypes. BEFV incidence in diseased cattle was 2.21 times higher than BTV despite the short viremia of BEFV.

An effective two-stage protocol was developed for BEFV viral isolation, when C6/36 cells were used in the first stage of VI, and the BSR cells were used as an irreplaceable cell line for the adaptation of the BEFV to cell lines of mammalian origin.

During 2015–2021, all BEF outbreaks in Israel were caused by local BEFV strains, whereas the epizootic of BEFV in 2023 was caused by a new “Mayotte-like” BEFV strain.

Phylogenetic analysis reveals the emergence of non-local BEFV strains not only in Israel but also in several Asian countries.

We suggested classifying BEFV strains according to phylogenetic, pairwise and proteomic analyses because previous classification based on the geographic origin of the viruses has lost its relevance. All BEFV strains can be classified as a single serotype subdivided into four lineages. Currently, each lineage varies from the others by more than 10% in nt sequences (about 12–13%), and the difference between sublineages IIIa and IIIb does not exceed 9%.

## Figures and Tables

**Table 1:** Information about field samples tested by RT-PCR for BEFV and BTV collected from clinical cattle in 2023 and the viral isolation in cell culture.

	Type of Samples				
	Blood	Int. Organs	Brain	Total	VI
№ of tested samples for BEFV	1128	39 (37)	9	1176 (1174)	17
№ of BEFV-positive samples	401	8	1	410	8
№ of tested samples for BTV	1070	39 (37)	9	1109 (1107)	25
№ of BTV-positive samples	118	6	1	125	11
№ of mixed positive BTV/BEFV samples	91	4	1 *	96	2 **
Total BTV positive samples	209	10	2	221	12
Total BEFV positive samples	492	12	2	506	9

№—number; blood—whole blood and serum samples; serum and whole blood were tested for BEFV; whole blood samples were tested for BTV; int. organs—internal organs which included spleen, lung or mixed samples (spleen and lung); VI—viral isolation; number in brackets—number of tested animals; *—the brain sample from a cow which manifested neural signs before death was also positive for the Shuni virus; **—one BTV and one BEFV, which were isolated from the same sample.

**Table 2:** Schematic procedure of the BTV and BEFV viral isolation (VI).

1st Step of VI			2nd Step of VI		
	BTV	BEFV		BTV	BEFV
ECE	+	-	C6/36 → BHK-21	NT	-
C6/36	+/-	+	C6/36 → BSR	+	+
BHK-21	NT	-	C6/36 → Vero	+/-	-
BSR	+/-	+/-	BSR → Vero	+	+
Vero	-	-			

+ successful VI; +/-—successful, but rare VI; -—unsuccessful VI; →—usage supernatant from successful VI (left side from the arrow) for the infection of other cell culture types (right side from the arrow); NT—not tested.

**Table 3:** Information about geographic locations with registered recurrent BEFV infection.

		Month of BEFV Detection						
Place	Distinct	Jun	Jul	Aug	Sep	Oct	Nov	Dec
BE’ER TOVIYYA	South	+	+	-	+	+	+	+
AZRIQAM	South	-	+	-	+	+	-	-
BENAYA	South	-	+	+	+	+	-	-
BET EL’AZARI	Center	-	-	-	+	+	-	-
GAL’ED (EVEN YIZHAQ)	North	-	-	-	+	-	+	-
HAMADYA	North	-	-	-	+	+	+	-
KEFAR MENAHEM	South	-	+	-	+	-	-	-
KEFAR WARBURG	South	-	+	-	+	-	+	-
LAVI	North	-	-	-	-	+	+	-
MASH’EN	South	-	+	-	+	-	-	-
MASSU’OT YIZHAQ	South	-	+	-	+	+	-	-
NIR YISRA’EL	South	-	+	-	+	-	-	-
NOV	North	-	-	-	-	+	+	+
QEVUZAT YAVNE	South	-	-	-	+	+	+	-
REGBA	North	-	-	-	+	-	+	-
SEDE YA’AQOV	North	-	-	+	+	+	-	-

+ laboratory-confirmed positive cases; - no laboratory-confirmed cases.

**Table 4:** Percentage identity of BEFV strains belonging to the same and different lineages.

Cluster	I	II	III	IIIa	IIIb	IV
I	98.78–98.83	86.7–87.69	87.51–87.94	87.51–87.88	87.57–87.94	86.34–86.74
II	86.70–87.69	96.53–98.66	86.94–88.01	87.03–87.29	86.94–88.01	86.92–87.64
III	87.51–87.94	86.94–88.01	91.13–99.73			89.30–91.89
IIIa	87.51–87.88	87.03–87.29		96.03–99.73	91.08–92.69	90.02–91.89
IIIb	87.57–87.94	86.94–88.01		91.08–92.69	94.93–98.18	89.30–91.34
IV	86.34–86.74	86.92–87.64	89.30–91.89	90.02–91.89	89.30–91.34	96.46–98.43

**Table 5:** Comparison of coding and noncoding regions of Israeli and global BEFV strains.

Strain	Origin	Lineage	N	ncr	P	ncr	M	ncr	G	ncr	Gns	ncr	α1	α2	β	ncr	γ	ncr	L
MN905763/IDR/2019	India	IIIa	431	36	279	57	223	61	623	63	586	45	89	116	147	49	104	40	2144
MN078236/2006/2000	Israel	IIIa	431	36	279	57	223	61	623	63	586	45	89	116	147	49	104	40	2144
OQ171224/ISR-1538/15/2015	Israel	IIIa	431	36	279	57	223	61	623	63	586	45	89	116	nd	nd	nd	nd	nd
OQ171223/ISR-1969/15/2015	Israel	IIIa	431	36	279	57	223	61	623	63	586	45	89	116	147	49	nd	nd	nd
OQ171226/ISR-2261/2/2018	Israel	IIIa	431	36	279	57	223	61	623	63	586	45	89	116	147	49	104	40	2144
OQ171225/ISR-2255/2018	Israel	IIIa	431	36	279	57	223	61	623	63	586	45	89	116	147	49	104	40	2144
PP239349/ISR-3096/1/2021	Israel	IIIa	431	36	279	57	223	61	623	63	586	45	89	116	147	49	104	40	2144
OQ171228/ISR-3180/1/2021	Israel	IIIa	431	36	279	57	223	61	623	63	586	45	89	116	147	49	104	40	2144
OQ171227/ISR-3119/2/2021	Israel	IIIa	431	36	279	57	223	61	623	63	586	45	89	116	147	49	104	40	2144
KM276084/Henan1/2012	China	IIIb	431	36	279	56	223	61	623	65	586	45	89	116	107	49	104	40	2144
AF234533/BB7721/1968	Australia	IV	431	36	279	56	223	61	623	65	586	45	89	116	107	49	104	40	2144
MN148803/7641/2017	Mayotte	I	431	35	279	52	223	60	623	73	586	36	89	116	147	49	104	55	2144
OR982678/ISR-1451/23	Israel	I	431	35	279	52	223	60	623	72	586	36	89	116	147	49	104	48	2144
OR982677/ISR-Negev/23	Israel	I	431	35	279	52	223	60	623	73	586	36	89	116	147	49	104	48	2144
OR982679/ISR-1520/23	Israel	I	431	35	279	52	223	60	623	72	586	36	89	116	147	49	104	48	2144
MW463337/RSA/OBP/2008	S.Africa	II	431	36	279	59	223	59	623	64	578	42	89	116	148	50	104	41	2144

ncr—noncoding region; S.Africa—South Africa; nd—no data (partial sequence). The coding regions of every protein are represented by the abbreviation of the protein.

## Data Availability

The original contributions presented in this study are included in the article/Supplementary Materials, and further inquiries can be directed to the corresponding authors.

## Supplementary Materials

The following supporting information can be downloaded at: https://www.mdpi.com/article/10.3390/pathogens13080636/s1, Figure S1: Phylogenetic tree of Israeli and global bovine ephemeral fever viruses (BEFVs) based on full-genome nucleotide sequences. Table S1: List of primers used for partial sequencing of local Israeli strains of bovine ephemeral fever virus.

## References

[1] Walker P.J. (2005). Bovine ephemeral fever in Australia and the world. Curr. Top. Microbiol. Immunol..

[2] Walker P.J., Klement E. (2015). Epidemiology and control of bovine ephemeral fever. Vet. Res..

[3] Li Z., Zheng F., Gao S., Wang S., Wang J., Liu Z., Du J., Yin H. (2015). Largescale serological survey of bovine ephemeral fever in China. Vet. Microbiol..

[4] Maiti S., Chakravarty P., Garai S., Bandyopadhyay S., Chouhan V.S. (2013). Ethno-veterinary practices for ephemeral fever in yak: A partipatory assessment by the Monpa tribe of Arunachal Pradesh. Indian J. Trad. Knowl..

[5] Malviya H.K., Prasad J. (1977). Ephemeral fever—A clinical and epidemiological study in cross bred cows and buffaloes. Indian Vet. J..

[6] McWilliam S.M., Kongsuwan K., Cowley J.A., Byrne K.A., Walker P.J. (1997). Genome organization and transcription strategy in the complex GNS-L intergenic region of bovine ephemeral fever rhabdovirus. J. Gen. Virol..

[7] Walker P.J., Byrne K.A., Cybinski D.H., Doolan D.L., Wang Y. (1991). Proteins of bovine ephemeral fever virus. J. Gen. Virol..

[8] Uren M.F., Walker P.J., Zakrzewski H., St George T.D., Byrne K.A. (1994). Effective vaccination of cattle using the virion G protein of bovine ephemeral fever virus as an antigen. Vaccine.

[9] Kongsuwan K., Cybinski D.H., Cooper J., Walker P.J. (1998). Location of neutralizing epitopes on the G protein of bovine ephemeral fever rhabdovirus. J. Gen. Virol..

[10] MDS Veterinary Manual. https://www.msdvetmanual.com/generalized-conditions/bovine-ephemeral-fever/bovine-ephemeral-fever.

[11] Dacheux L., Dommergues L., Chouanibou Y., Doméon L., Schuler C., Bonas S., Luo D., Maufrais C., Cetre-Sossah C., Cardinale E. (2019). Co-circulation and characterization of novel African arboviruses (genus *Ephemerovirus*) in cattle, Mayotte island, Indian Ocean, 2017. Transbound. Emerg. Dis..

[12] Barigye R., Davis S., Hunt R., Hunt N., Walsh S., Elliott N., Burnup C., Aumann S., Day C., Dyrting K. (2016). Viral neurotropism, peripheral neuropathy and other morphological abnormalities in bovine ephemeral fever virus-infected downer cattle. Aust. Vet. J..

[13] Hill M.W., Schultz K. (1977). Ataxia and paralysis associated with bovine ephemeral fever infection. Aust. Vet. J..

[14] Zheng F., Qiu C. (2012). Phylogenetic relationships of the glycoprotein gene of bovine ephemeral fever virus isolated from mainland China, Taiwan, Japan, Turkey, Israel and Australia. Virol. J..

[15] Hsieh Y.C., Chen S.H., Chou C.C., Ting L.J., Itakura C., Wang F.I. (2005). Bovine ephemeral fever in Taiwan (2001–2002). J. Vet. Med. Sci..

[16] Tonbak S., Berber E., Yoruk M.D., Azkur A.K., Pestil Z., Bulut H. (2013). A large-scale outbreak of bovine ephemeral fever in Turkey, 2012. J. Vet. Med. Sci..

[17] Yeruham I., Van Ham M., Stram Y., Friedgut O., Yadin H., Mumcuoglu K.Y., Braverman Y. (2010). Epidemiological investigation of bovine ephemeral Fever outbreaks in Israel. Vet. Med. Int..

[18] Lavon Y., Ezra E., Friedgut O., Behar A. (2023). Economic Aspects of Bovine Ephemeral Fever (BEF) Outbreaks in Dairy Cattle Herds. Vet. Sci..

[19] Zheng F.Y., Chen Q.W., Li Z., Gong X.W., Wang J.D., Yin H. (2016). Experimental infection with bovine ephemeral fever virus and analysis of its antibody response cattle. Res. Vet. Sci..

[20] Bevan L.E.W. (1907). Preliminary report on the so-called stiff-sickness or 3-day-sickness of cattle. J. Comp. Path..

[21] Nandi S., Negi B.S. (1999). Bovine ephemeral fever: A review. Comp. Immunol. Microbiol. Infect. Dis..

[22] Ragbagliati D.S. (1924). Three day’s fever or stiff sickness in cattle. Vet. Rec..

[23] Sen S.K. (1931). Three-day sickness of cattle. Ind. J. Vet. Sci..

[24] Rosen S. (1931). Ephemeral fever (three days’ fever) of cattle in Palestine. Vet. J..

[25] Burgess G.W. (1971). Bovine ephemeral fever: A review. Vet. Bull..

[26] Abu Elzein E.M.E., Gameel A.A., Al Afaleq A.I., Al Gundi O., Bukhari A. (1997). Bovine ephemeral fever in Saudi Arabia. Vet. Record..

[27] Karayel-Hacioglu I., Duran S., Yelken Y., Vezir Y., Unal N., Alkan F. (2021). Isolation and genetic characterization of bovine ephemeral fever virus from epidemic-2020 in Turkey. Trop. Anim. Health. Prod..

[28] Pekmez K., Kaplan M., Çağırgan A., Arslan F. (2024). The origin and molecular characterization of the BEF virus causing the small-scale epidemic in Western Turkey. Authorea.

[29] Almasi S., Bakhshesh M. (2019). Antigenic variation of bovine ephemeral fever viruses isolated in Iran, 2012–2013. Virus Genes.

[30] Rezatofighi S.E., Mirzadeh K., Mahmoodi F. (2022). Molecular characterization and phylogenetic analysis of bovine ephemeral fever viruses in Khuzestan province of Iran in 2018 and 2020. BMC Vet. Res..

[31] Zaher S., Ahmed W. (2011). Investigations on Bovine Ephemeral fever virus in Egyptian cows and buffaloes with emphasis on isolation and identification of a field strain. Glob. Vet..

[32] Aziz-Boaron O., Klausner Z., Hasoksuz M., Shenkar J., Gafni O., Gelman B., David D., Klement E. (2012). Circulation of bovine ephemeral fever in the Middle East—Strong evidence for transmission by winds and animal transport. Vet. Microbiol..

[33] The WOAH Website. https://www.woah.org/fileadmin/Home/eng/Health_standards/tahm/3.01.03_BLUETONGUE.pdf.

[34] The WOAH Website. https://www.woah.org/en/disease/bluetongue/#:~:text=BT%20is%20a%20disease%20listed,OIE%20Terrestrial%20Animal%20Health%20Code).

[35] Royal D.G. Ahead in Animal Health. https://www.gdanimalhealth.com/en/News/2024/01/Highlights-report-cattle-december-2023.

[36] Golender N., Klement E., Kovtunenko A., Even-Tov B., Zamir L., Tiomkin E., Kenigswald G., Hoffmann B. (2023). Comparative Molecular and Epidemiological Analyses of Israeli Bluetongue Viruses Serotype 1 and 9 Causing Outbreaks in 2018–2020. Microorganisms.

[37] Golender N., Eldar A., Ehrlich M., Kenigswald G., Shlamovitz I., Even-Tov B., Zamir L., Klement E., Bumbarov V. (2021). Genomic Analysis Illustrated a Single Introduction and Evolution of Israeli Bluetongue Serotype 8 Virus Population 2008–2019. Microorganisms.

[38] Stewart M., Hardy A., Barry G., Pinto R.M., Caporale M., Melzi E., Hughes J., Taggart A., Janowicz A., Varela M. (2015). Characterization of a second open reading frame in genome segment 10 of bluetongue virus. J. Gen. Virol..

[39] Ries C., Sharav T., Tseren-Ochir E.O., Beer M., Hoffmann B. (2020). Putative Novel Serotypes ‘33’ and ‘35’ in Clinically Healthy Small Ruminants in Mongolia Expand the Group of Atypical BTV. Viruses.

[40] Ries C., Vögtlin A., Hüssy D., Jandt T., Gobet H., Hilbe M., Burgener C., Schweizer L., Häfliger-Speiser S., Beer M. (2021). Putative Novel Atypical BTV Serotype ‘36’ Identified in Small Ruminants in Switzerland. Viruses.

[41] Golender N., Hoffmann B. (2024). The Molecular Epidemiology of Epizootic Hemorrhagic Disease Viruses Identified in Israel between 2015 and 2023. Epidemiologia.

[42] Komarov A., Goldsmit L. (1951). A disease similar to Blue Tongue in cattle and sheep in Israel. Ref. Vet..

[43] Erster O., Stram R., Menasherow S., Rubistein-Giuni M., Sharir B., Kchinich E., Stram Y. (2017). High-resolution melting (HRM) for genotyping bovine ephemeral fever virus (BEFV). Virus Res..

[44] Golender N., Klement E., Ofer L., Hoffmann B., Wernike K., Beer M., Pfaff F. (2023). Hefer valley virus: A novel ephemerovirus detected in the blood of a cow with severe clinical signs in Israel in 2022. Arch. Virol..

[45] Wernike K., Hoffmann B., Beer M. (2015). Simultaneous detection of five notifiable viral diseases of cattle by single-tube multiplex real-time RT-PCR. J. Virol. Methods.

[46] Golender N., Bumbarov V.Y., Erster O., Beer M., Khinich Y., Wernike K. (2018). Development and validation of a universal S-segment-based real-time RT-PCR assay for the detection of Simbu serogroup viruses. J. Virol. Methods.

[47] Kumar S., Stecher G., Li M., Knyaz C., Tamura K. (2018). MEGA X: Molecular Evolutionary Genetics Analysis across Computing Platforms. Mol. Biol. Evol..

[48] Alkan F., Albayrak H., Timurkan M.O., Ozan E., Coskun N. (2017). Assessment of the molecular epidemiology of bovine ephemeral fever in Turkey. Vet. Arh..

[49] Kalonda A., Saasa N., Kajihara M., Nao N., Moonga L., Ndebe J., Mori-Kajihara A., Mukubesa A.N., Sakoda Y., Sawa H. (2024). Phylogenetic Analysis of Newcastle Disease Virus Isolated from Poultry in Live Bird Markets and Wild Waterfowl in Zambia. Microorganisms.

[50] de Martinis C., Cardillo L., Pesce F., Viscardi M., Cozzolino L., Paradiso R., Cavallo S., De Ascentis M., Goffredo M., Monaco F. (2023). Reoccurrence of West Nile virus lineage 1 after 2-year decline: First equine outbreak in Campania region. Front. Vet. Sci..

[51] Wang P.H., Shah P.T., Xing L. (2023). Genetic characteristics and geographic distribution of rabies virus in China. Arch. Virol..

[52] Biguezoton A.S., Ilboudo G.S., Wieland B., Sawadogo R.W., Dah F.F., Sidibe C.A.K., Zoungrana A., Okoth E., Dione M. (2024). Molecular Epidemiology of Peste Des Petits Ruminants Virus in West Africa: Is Lineage IV Replacing Lineage II in Burkina Faso?. Viruses.

[53] Mackerras I.M., Mackerras M.J., Burnet F.M. (1940). Experimental studies of ephemeral fever in Australian cattle. CSIRO Bull..

[54] Hilke J., Strobel H., Woelke S., Stoeter M., Voigt K., Moeller B., Bastian M., Ganter M. (2019). Presence of Antibodies against Bluetongue Virus (BTV) in Sheep 5 to 7.5 Years after Vaccination with Inactivated BTV-8 Vaccines. Viruses.

[55] Gleser D., Spinner K., Klement E. (2023). Effectiveness of the strain 919 bovine ephemeral fever virus vaccine in the face of a real-world outbreak: A field study in Israeli dairy herds. Vaccine.

[56] Gleser D., Cohen M., Kenigswald G., Kedmi M., Sharir B., Klement E. (2024). Optimizing Protocols for the 919 Strain Based Bovine Ephemeral Fever Virus Vaccine: Evaluation of Dose-Dependent Effectiveness and Long-Term Immunity. Vaccine.

[57] Aziz-Boaron O., Leibovitz K., Gelman B., Kedmi M., Klement E. (2013). Safety, immunogenicity and duration of immunity elicited by an inactivated bovine ephemeral fever vaccine. PLoS ONE.

[58] Gasparini M., Laguardia-Nascimento M., Sales É.B., Oliveira A.G.G., Lobato Z.I.P., Camargos M.F., Fonseca Júnior A.A. (2021). Study of molecular diagnosis and viremia of bluetongue virus in sheep and cattle. Braz. J. Microbiol..

[59] St George T.D., Ryan P., Aaskov J., Russell R. (2009). Evidence that mosquitoes are the vectors of bovine ephemeral fever virus. Book Arbovirus Research in Australia.

