# Supplementary material for: Bovine Ephemeral Fever Viruses in Israel 2014–2023: Genetic Characterization of Local and Emerging Strains
Source: Pathogens. 2024 Jul 29;13(8):636. doi: 10.3390/pathogens13080636 (PMC11357334; doi:10.3390/pathogens13080636)
Supplement: Supplementary file 1 [file pathogens-13-00636-s001.zip › pathogens-3074599-supplementary.pdf]

Table S1. List of primers used for partial sequencing of local Israeli strains of bovine ephemeral fever virus

name	Oligo sequence (5′ to 3′)	length of product	source
BEF-37F	ACC CAA TTA GTG TTT TAA CAG GTC	869	this study
BEF-883R	CCA TCA TAT ATG GCA TGT AAG AG		this study
BEF-789F	CCA TTG AGG AGA TTC TTG ATT G	885	this study
BEF-1651R	AGT AAG CTG TTT TCC CAG TCT TC		this study
BEF-1579F	ATT AAA CAA GAA CCT GGC ATT TAC	857	this study
BEF-2415R	ATA ATC CGG AGG AGC TGT ACC		this study
BEF-2334F	TTC AAG AAA GGG AAG TCC AAA G	723	this study
BEF-3033R	GGT CCT GTT AAC CCA ACT CTA TTT		this study
BEF-2945F	TTA TAA TTT GAA GGG AGA GCA CG	869	this study
BEF-3792R	CCA CTT CTT CCA CAA AAT GTA G		this study
BEF-3676F	TAG TAA RCA TTG GGA ATG CAT CAC	906	this study
BEF-4559R	TTA ATT TGA CTT GTT TCA TGT GG		this study
BEF-4467F	TGA TCA AAT TTG AGG TAA TGG A	983	this study
BEF-5428R	ATT CAA GCT CGC TAA YAG AAG A		this study
BEF-5287F	AAT TTC GAT CAT GAG GTA ACA TA	971	this study
BEF-6235R	GGA TTA GCC TGT TCT TTA TAC CA		this study
BEF-6142F	GGG ATT AAA AAG AAT TTA ACT GAC G	634	this study
BEF-6754R	TTC ATG MTC AAT AAT CCA ACT T		this study
			this study
			this study

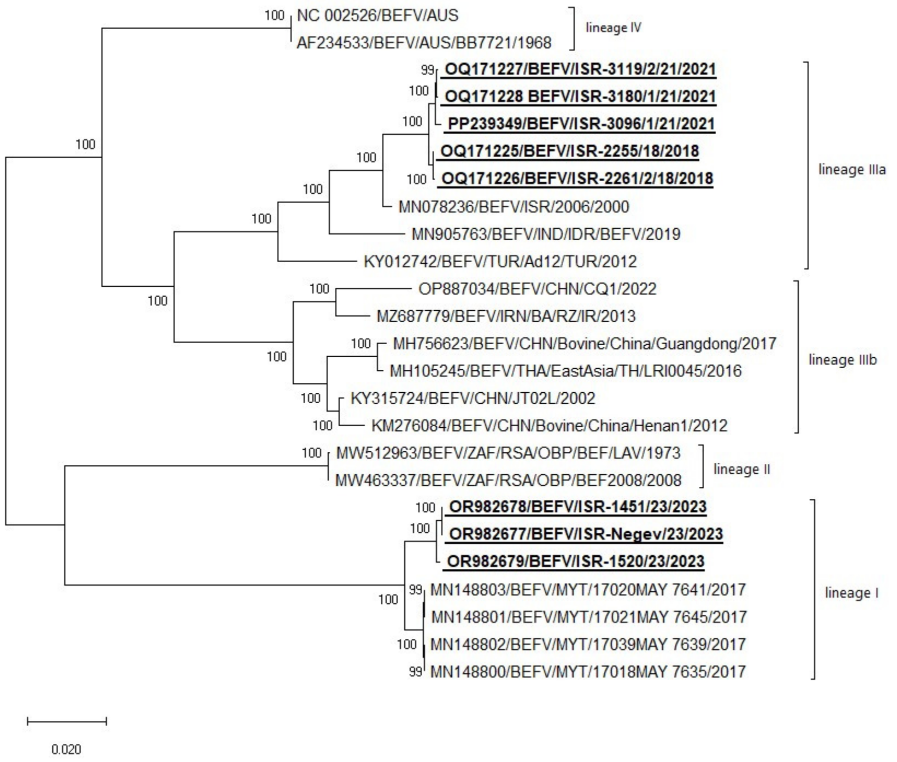

Figure S1. Phylogenetic tree of Israeli and global bovine ephemeral fever viruses (BEFV) basing on full genome nucleotide sequences. All Israeli BEFV strains shown in bold, while strains sequenced during the current study, are signed in bold and underlined. The phylogeny was inferred using the Maximum Likelihood method and Tamura-Nei model method. The percentage of replicate trees in which the associated taxa clustered together in the bootstrap test (1000 replicates) are shown next to the branches. Viruses were identified by accession number/virus species/location/isolate/year.
